# First-in-human pilot trial of combined intracoronary and intravenous mesenchymal stem cell therapy in acute myocardial infarction

**DOI:** 10.3389/fcvm.2022.961920

**Published:** 2022-08-09

**Authors:** Lien-Cheng Hsiao, Yen-Nien Lin, Woei-Cherng Shyu, Ming Ho, Chiung-Ray Lu, Shih-Sheng Chang, Yu-Chen Wang, Jan-Yow Chen, Shang-Yeh Lu, Mei-Yao Wu, Keng-Yuan Li, Yu-Kai Lin, Wen-Yih I. Tseng, Mao-Yuan Su, Chin-Ting Hsu, Cheng-Kang Tsai, Lu-Ting Chiu, Chien-Lin Chen, Cheng-Li Lin, Kai-Chieh Hu, Der-Yang Cho, Chang-Hai Tsai, Kuan-Cheng Chang, Long-Bin Jeng

**Affiliations:** ^1^Division of Cardiovascular Medicine, Department of Medicine, China Medical University Hospital, Taichung, Taiwan; ^2^School of Medicine, China Medical University, Taichung, Taiwan; ^3^Department of Neurology and Translational Medicine Research Center, China Medical University Hospital, Taichung, Taiwan; ^4^Ever Supreme Bio Technology Co., Ltd, Taichung, Taiwan; ^5^Department of Obstetrics and Gynecology, China Medical University Hospital, Taichung, Taiwan; ^6^School of Post-Baccalaureate Chinese Medicine, China Medical University, Taichung, Taiwan; ^7^Department of Chinese Medicine, China Medical University Hospital, Taichung, Taiwan; ^8^Molecular Imaging Center, National Taiwan University, Taipei, Taiwan; ^9^Management Office for Health Data, China Medical University Hospital, Taichung, Taiwan; ^10^Department of Neurosurgery, China Medical University Hospital, Taichung, Taiwan; ^11^Organ Transplantation Center, China Medical University Hospital, Taichung, Taiwan

**Keywords:** intracoronary, intravenous, umbilical mesenchymal stem cell, acute myocardial infarction, human pilot trial

## Abstract

**Background:**

Acute ST-elevation myocardial infarction (STEMI) elicits a robust cardiomyocyte death and inflammatory responses despite timely revascularization.

**Objectives:**

This phase 1, open-label, single-arm, first-in-human study aimed to assess the safety and efficacy of combined intracoronary (IC) and intravenous (IV) transplantation of umbilical cord-derived mesenchymal stem cells (UMSC01) for heart repair in STEMI patients with impaired left ventricular ejection fraction (LVEF 30-49%) following successful reperfusion by percutaneous coronary intervention.

**Methods:**

Consenting patients received the first dose of UMSC01 through IC injection 4-5 days after STEMI followed by the second dose of UMSC01 via IV infusion 2 days later. The primary endpoint was occurrence of any treatment-related adverse events and the secondary endpoint was changes of serum biomarkers and heart function by cardiac magnetic resonance imaging during a 12-month follow-up period.

**Results:**

Eight patients gave informed consents, of whom six completed the study. None of the subjects experienced treatment-related serious adverse events or major adverse cardiovascular events during IC or IV infusion of UMSC01 and during the follow-up period. The NT-proBNP level decreased (1362 ± 1801 vs. 109 ± 115 pg/mL, *p* = 0.0313), the LVEF increased (52.67 ± 12.75% vs. 62.47 ± 17.35%, *p* = 0.0246), and the wall motion score decreased (26.33 ± 5.57 vs. 22.33 ± 5.85, *p* = 0.0180) at the 12-month follow-up compared to the baseline values. The serial changes of LVEF were 0.67 ± 3.98, 8.09 ± 6.18, 9.04 ± 10.91, and 9.80 ± 7.56 at 1, 3, 6, and 12 months, respectively as compared to the baseline.

**Conclusion:**

This pilot study shows that combined IC and IV transplantation of UMSC01 in STEMI patients with impaired LVEF appears to be safe, feasible, and potentially beneficial in improving heart function. Further phase 2 studies are required to explore the effectiveness of dual-route transplantation of UMSC01 in STEMI patients.

## Graphical Abstract

Eligible STEMI patients with a LVEF of 30-49% received the first dose of UMSC01 through IC injection 4-5 days after STEMI and the second dose of UMSC01 via IV infusion 2 days after IC. The culprit arteries were patent with comparable FEV1% and decreases in cardiac enzymes after IC + IV infusion. The LVEF increased at 12-month follow-up by cardiac MRI. 
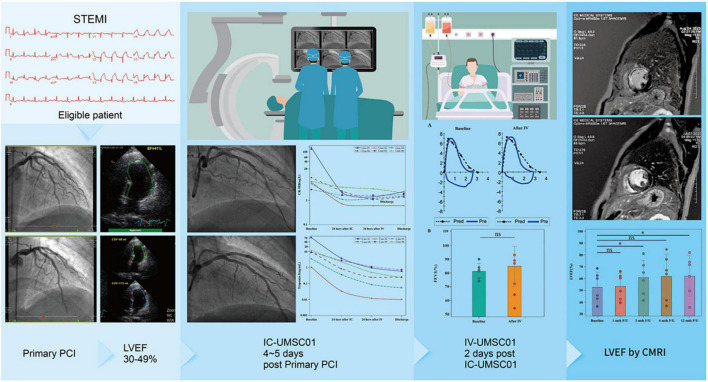

## Introduction

Acute myocardial infarction (AMI) elicits a robust cardiomyocyte death despite timely revascularization and optimal treatment (1). Acute injury initiates substantial inflammatory response, which causes cardiomyocyte damage and triggers cardiac fibrosis (2). Limitation of the initial injury and appropriate regulation of the immune response are essential to improve healing and reduce unfavorable left ventricular remodeling (3, 4). Stem cell-based therapies have been proposed as a promising strategy to reduce cardiomyocyte death and modulate immune reactions, leading to reduced myocardial scarring and improved cardiac function regardless of ischemic or non-ischemic cardiomyopathy (5–8).

Bone marrow mononuclear cells (BMNCs) and circulating progenitor/stem cells delivered via the intracoronary (IC) or intravenous (IV) route constitute the most commonly used cell types, with doses ranging between 10^6^ and 10^9^ in published clinical studies (9–12). Recently, mesenchymal stem cells (MSCs) have drawn much attention because of their anti-inflammatory and immunomodulatory effects (13–16), and their effectiveness has been documented in many clinical trials (17, 18).

Previous studies in animals and humans have suggested that the dose of transplanted cells plays an important role in determining eventual myocardial function indices (19). Delivering sufficient cells by repeated transplantation might be necessary to overcome low retention and survival rates in the infarcted myocardium (7, 19). It has also been shown that three repeated doses of cells are superior to one dose of equivalent number of cells in relation to LVEF improvement, possibly due to greater antifibrotic and anti-inflammatory actions (20). There are various strategies regarding repeated transplantation, which involves the different combinations including deliver method, time interval and cell dose. Yao et al. demonstrated that repeated IC infusion of BMNCs 3 months after the first transfer is feasible and beneficial in patients with a large AMI (19). However, there has been no study in evaluating the feasibility and safety by combing IC and IV administration to achieve a sufficiently higher dose of umbilical cord-derived MSCs in patients with AMI.

To enhance the effect of cell-based therapy in post-AMI cardiac repair, we investigated umbilical-MSCs as a therapeutic candidate. Although neither IC nor IV transplantation are ideal, they can complement each other (21). We hypothesized that a combined IC and subsequent IV umbilical-MSCs transplantation enables a synergistic anti-inflammatory and direct cell repair effect, which is beneficial in patients with AMI. Here, we first assessed the safety and explored the preliminary efficacy of umbilical cord-derived MSCs (UMSC01) by combining IC and IV stem cell administration.

## Materials and methods

### Study design and patients

**FIGURE 1:**
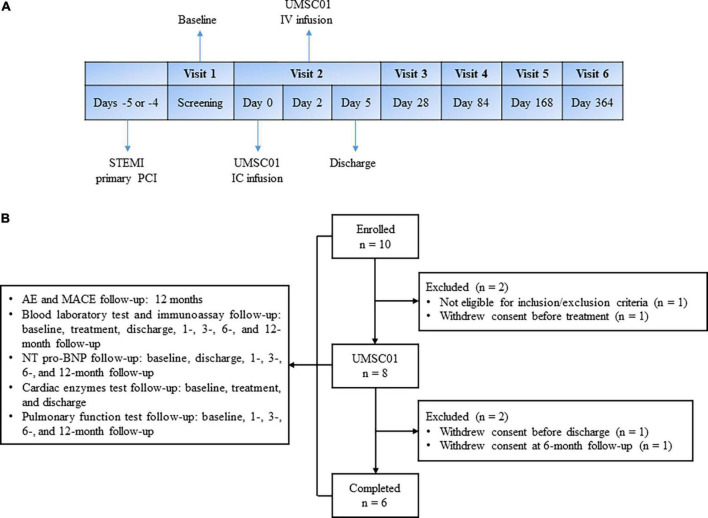
Study design and subject disposition. **(A)** Eligible patients who presented with first-ever ST elevation myocardial infarction (STEMI) were consecutively recruited. All of the subjects were hospitalized from the day of primary percutaneous coronary intervention (PCI), until the third day after IV infusion of UMSC01. IC infusion of UMSC01 was performed 4–5 days via the index culprit artery after successful revascularization. The day of IC infusion of UMSC01 was designed as Day 0 and IV infusion of UMSC01 was carried out on Day 2. After discharge, all subjects were followed up at 1, 3, 6, and 12 months for endpoint evaluation. **(B)** Between August 20, 2019, and August 2, 2020, we screened 10 patients with STEMI, of whom eight eligible patients provided written informed consent to participate in the clinical trial (NCT04056819). Two subjects withdrew consent during the follow-up period. Six subjects were followed up for the primary and secondary endpoints at 12 months. AE, adverse event; MACE, major adverse cardiovascular events; NT-pro-BNP, amino-terminal pro-brain natriuretic peptide.

This was a phase one, open-label, single-arm, single- center study in patients with acute ST-elevation MI (STEMI). Eligible patients who presented with first-ever STEMI were consecutively recruited. All of the subjects were hospitalized from the day of primary percutaneous coronary intervention (PPCI), until the third day after IV infusion of UMSC01. IC infusion of UMSC01 was performed 4–5 days via the index culprit artery after successful revascularization. The day of IC infusion of UMSC01 was designed as Day 0 and IV infusion of UMSC01 was carried out on Day 2. After discharge, all subjects were followed up at 1, 3, 6, and 12 months for endpoint evaluation, as shown in Figure 1A.

Male or female, aged between 20 and 76 years who presented with typical ischemic chest pain within 12 h of symptom onset and were diagnosed with first-time acute STEMI were considered eligible for the study. The infarct-related artery should be successfully revascularized by PPCI with a thrombolysis in myocardial infarction (TIMI) flow ≥ 2 suitable for cell infusion. After revascularization, STEMI patients should fulfill an echocardiography-determined left ventricular ejection fraction (LVEF) of ≥30% and <50% for enrollment. Patients with profound cardiogenic shock requiring mechanical support or those who had previous or incident significant valvular heart diseases were excluded. The full list of the inclusion and exclusion criteria can be found in online Supplementary material.

This study complied with the ethical principles of Declaration of Helsinki and was approved by the Research Ethics Committee of China Medical University Hospital (CMUH107-REC1-088). The trial was monitored by an independent data and safety monitoring board who met as planned to assess adverse events.

### Preparation of UMSC01

On the day before administration, the cryopreserved UMSC01 cells were thawed in a 37°C water bath. The cell suspension was washed three times to minimize residual reagents during the manufacturing process. The cell pellet was then resuspended in normal saline at a final cell density of ∼1 × 10^7^ cells/mL for administration. The cell identity test showed that ≥95% of cells expressed CD73, CD90, and CD105, while the expression level of CD11b, CD19, CD45, CD34, and human leukocyte antigen-DR isotype (HLA-DR) was ≤2%. The final volume of UMSC01 for infusion (drug product) was resuspended in 0.9% saline containing a cell dose of either 1 × 10^7^ for IC or 9 × 10^7^ for IV infusion in AT-Closed Vial (Aseptic Technology, Gembloux, Belgium), while fulfilling the following product release criteria: cell viability by trypan blue (>70%), purity test by endotoxin examination (<0.25 EU/mL), and sterility test by gram staining and direct inoculation. The stepwise preparation of UMSC01 cells was described in the Supplementary Methods.

### Intracoronary administration of UMSC01

We used the stop-flow technique for IC injection of UMSC01 with positioning of an over-the-wire balloon catheter within the segment of the previously deployed stent in the infarct-related artery (22). Briefly, 1 × 10^7^ of UMSC01 in packed AT-Closed Vial^®^ was mixed in 14 mL normal saline with 10,000 U/L heparin and directly injected using the over-the-wire balloon catheter in three treatment cycles. Each treatment cycle included 1.5 min of balloon inflation for cell injection, followed by 1.5 min balloon deflation for coronary reperfusion. Three cycles of cell injection were completed within 15 min. After completion of cell infusion, coronary angiography was repeated to confirm the patency of coronary artery flow. Then, patients were transferred to the intensive care unit for overnight observation. Blood pressures, heart rate, oxygenation, and ECGs were continuously monitored throughout the procedure.

### Intravenous administration of UMSC01

For IV infusion of UMSC01, 9 × 10^7^ of UMSC01 mixed in 141 mL normal saline with 10,000 U/L heparin was administered via an antecubital vein at a flow rate of 2.5 mL/minute within 60 min. Blood pressures, heart rate, oxygenation, and ECGs were closely monitored throughout the procedure. Pulmonary function test with a ratio of the first second of forced expiration to the forced vital capacity (FEV1%) was measured before and 24 h after IV infusion. After completing the IV infusion of UMSC01, patients were transferred to the intensive care unit for overnight observation.

### Study endpoints

The primary endpoints for the study were emergence of any suspected or unexpected serious adverse reaction and occurrence of major adverse cardiovascular events (MACE) including death, recurrent AMI, stroke, and target-vessel revascularization. The secondary endpoints were changes of the serum level of amino-terminal pro-brain natriuretic peptide (NT-proBNP) from baseline to discharge and at 1-, 3-, 6-, and 12-month follow-up and left ventricular function evaluated by cardiac magnetic resonance imaging (CMRI) from baseline to 1-, 3-, 6-, and 12-month follow-up.

### Statistical analyses

All analyses were performed on patients who completed the entire study from the time of screening to the 12-month follow-up. Continuous variables are presented as mean and standard deviation, and categorical variables are presented as frequency and percentage. To compare the differences between baseline and follow-ups, paired *t*-tests were performed for efficacy endpoints to have more statistical power when the normality assumptions were not violated. Wilcoxon signed-rank tests were used for safety endpoints or non-normally distributed data. The Shapiro–Wilk method was adopted for normality tests because of small sample sizes. A two-sided *p*-value < 0.05 was considered statistically significant. All statistical analyses were performed using SAS software, version 9.4 (SAS Institute, Cary, NC, United States).

## Results

### Subject disposition

Between August 20, 2019, and August 2, 2020, we screened 10 patients with ST-elevation AMI, of whom eight eligible patients provided written informed consent to participate in the clinical trial (NCT04056819). Two subjects withdrew consent during the follow-up period. Six subjects were followed up for the primary and secondary endpoints at 12 months. The subject disposition is shown in Figure 1B.

### Patient characteristics

Baseline characteristics of the study subjects are shown in Table 1. All the enrolled subjects were males with a mean age of 61 ± 10 years. All the infarct-related arteries (left anterior descending artery in four patients and right coronary artery in two patients) underwent successful revascularization with TIMI 3 flow. The mean LVEF by echocardiography after PPCI was 43.0 ± 2.7 (range, 39.9-46.3%), which was associated with a baseline New York Heart Association functional class of II in all study subjects. None of the subjects had significant valvular heart disease, new-onset atrial fibrillation, or mechanical ventilation support. Following successful PPCI revascularization, all patients received guideline-directed medical treatment including dual-antiplatelet, β-blockers, angiotensin-converting enzyme inhibitors/angiotensin II receptor blockers, and statins.

**TABLE 1:** Baseline characteristics of the study subjects.

.	Patient 1	Patient 2	Patient 3	Patient 4	Patient 5	Patient 6	Mean ± SD/n (%)
**Age (year)**	53	48	70	71	56	70	61 ± 10
**Gender**							
Male	**Y**	**Y**	**Y**	**Y**	**Y**	**Y**	6 (100.00)
**Height (cm)**	166	180	164	165	178	165	170 ± 7
**Weight (kg)**	83	78	58	61	96	62	73 ± 15
**BMI (kg/m^2^)**	30.1	24.1	21.6	22.4	30.3	22.8	25.2 ± 3.9
**Smoking**	**Y**	**Y**	EX*^a^*	**Y**	**Y**	**Y**	5 (83.33)
**Vital signs**							
Systolic blood pressure (mmHg)	141	130	132	127	115	90	123 ± 18
Diastolic blood pressure (mmHg)	89	75	77	74	79	61	76 ± 9
Heart rate (bpm)	91	75	58	49	81	99	76 ± 19
Oximetry (%)	–	95	100	100	100	100	99 ± 2
**Medical history**							
Type 2 diabetes mellitus	N	N	N	N	**Y**	N	1 (16.67)
Hypertension	N	**Y**	**Y**	**Y**	**Y**	N	4 (66.67)
Dyslipidemia	**Y**	**Y**	**Y**	N	**Y**	N	4 (66.67)
Old myocardial infarction	N	N	N	N	N	N	0 (0.00)
Prior heart failure	N	N	N	N	N	N	0 (0.00)
Coronary artery disease	N	N	**Y**	N	N	N	1 (16.67)
ESRD	N	N	N	N	N	N	0 (0.00)
Stroke	N	N	N	N	N	N	0 (0.00)
COPD	N	N	**Y**	N	N	N	1 (16.67)
Liver cirrhosis	N	N	N	N	N	N	0 (0.00)
Cancer	N	N	N	N	N	N	0 (0.00)
**Infarct-related artery**							
LM	N	N	N	N	N	N	0 (0.00)
LAD	**Y**	**Y**	N	N	**Y**	**Y**	4 (66.67)
LCX	N	N	N	N	N	N	0 (0.00)
RCA	N	N	**Y**	**Y**	N	N	2 (33.33)
**Culprit vessel TIMI flow after PCI**							
0	N	N	N	N	N	N	0 (0.00)
1	N	N	N	N	N	N	0 (0.00)
2	N	N	N	N	N	N	0 (0.00)
3	**Y**	**Y**	**Y**	**Y**	**Y**	**Y**	6 (100.00)
**Killip classification**							
I	**Y**	N	**Y**	**Y**	**Y**	**Y**	5 (83.33)
II	N	N	N	N	N	N	0 (0.00)
III	N	N	N	N	N	N	0 (0.00)
IV	N	**Y**	N	N	N	N	1 (16.67)
**NYHA functional classification**							
I	N	N	N	N	N	N	0 (0.00)
II	**Y**	**Y**	**Y**	**Y**	**Y**	**Y**	6 (100.00)
III	N	N	N	N	N	N	0 (0.00)
IV	N	N	N	N	N	N	0 (0.00)
**LVEF (%) by echocardiography after PCI**	40.7	42.5	46.0	46.3	39.9	42.6	43.0 ± 2.7
**Significant valvular heart disease**	N	N	N	N	N	N	0 (0.00)
**New onset atrial fibrillation**	N	N	N	N	N	N	0 (0.00)
**Laboratory test at emergency room**							
AST (IU/L)	116	13	16	13	52	15	38 ± 41
Creatinine (mg/dL)	0.95	1.59	1.05	1.14	0.91	1.19	1.14 ± 0.25
eGFR (mL/min/1.73 m^2^)	83	47	70	63	86	60	68 ± 15
Sodium (mmol/L)	138	140	139	141	140	143	140 ± 2
Potassium (mmol/L)	3.3	3.9	3.8	4.1	4.0	4.2	3.9 ± 0.3
RBC (10^6^/μL)	6.04	–	–	5.38	5.58	4.88	5.47 ± 0.48
WBC (10^3^/μL)	13.7	18.2	8.2	15.0	10.5	5.3	11.8 ± 4.7
Hemoglobin (g/dL)	17.6	16.2	14.5	15.4	16.6	16.4	16.1 ± 1.1
Platelet count (10^3^/μL)	219	288	213	233	249	251	242 ± 27
Troponin-I (ng/mL)	0.3769	0.2800	<0.0100	0.0371	<0.0100	0.2808	0.2437 ± 0.1451
**Medications**							
Aspirin	**Y**	**Y**	**Y**	**Y**	**Y**	**Y**	6 (100.00)
Clopidogrel	N	**Y**	N	N	N	N	1 (16.67)
Ticagrelor	**Y**	N	**Y**	**Y**	**Y**	**Y**	5 (83.33)
Glycoprotein IIb/IIIa inhibitors	N	N	N	**Y**	**Y**	N	2 (33.33)
Heparin	**Y**	**Y**	**Y**	**Y**	**Y**	**Y**	6 (100.00)
Beta blockers	**Y**	**Y**	**Y**	**Y**	**Y**	**Y**	6 (100.00)
ACEI/ARB	**Y**	**Y**	**Y**	**Y**	**Y**	**Y**	6 (100.00)
MRA	N	N	N	N	N	N	0 (0.00)
Statins	**Y**	**Y**	**Y**	**Y**	**Y**	**Y**	6 (100.00)
Vasopressor	N	N	N	N	N	N	0 (0.00)
**Mechanical ventilation**	N	N	N	N	N	N	0 (0.00)
**IABP**	N	**Y**	N	N	N	N	1 (16.67)

^a^Ex-smoker.

ACEI, angiotensin converting enzyme inhibitor; ARB, angiotensin ii receptor blocker; AST, aspartate aminotransferase; BMI, body mass index; COPD, chronic obstructive pulmonary disease; eGFR, estimated glomerular filtration rate; ESRD, end-stage renal disease; IABP, intra-aortic balloon pump; LAD, left anterior descending artery; LCX, left circumflex artery; LM, left main; LVEF, left ventricular ejection fraction; MRA, mineralocorticoid receptor antagonist; NYHA, New York heart association; PCI, percutaneous coronary intervention; RBC, red blood cell; RCA, right coronary artery; SD, standard deviation; TIMI, thrombolysis in myocardial infarction; WBC, white blood cell.

### Characterization of UMSC01

**FIGURE 2:**
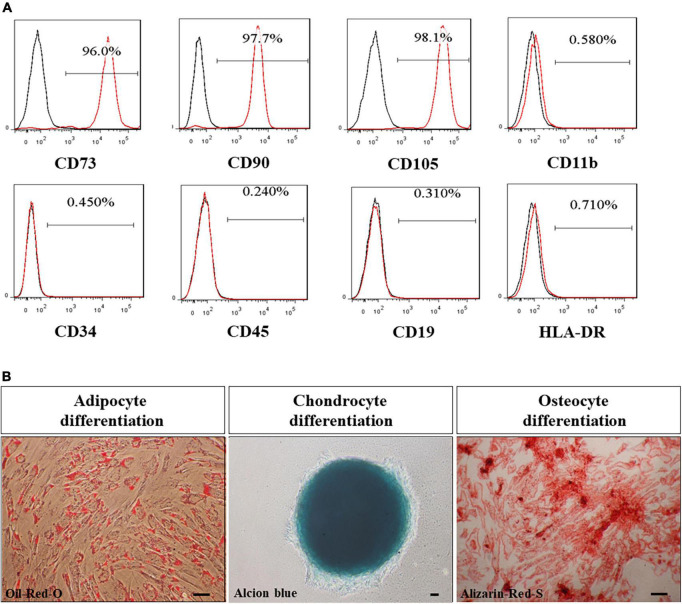
Characterization of human umbilical cord-derived mesenchymal stem cells (UMSC01). **(A)** The clinical grade of UMSC01 was certified and identified by surface markers, which showed uniformly high expression (≥95%) of CD73, CD90, and CD105 but low expression (≤2%) of CD11b, CD34, CD45, CD19, and HLA-DR. **(B)** The potency assay for the capacity of *in vitro* differentiation into mesodermal lineages of adipocytes, chondrocytes, and osteocytes was confirmed by Oil-Red-O, Alcian-Blue and Alizarin-Red staining, respectively. Bar = 50 μm. CD, cluster of differentiation; HLA, human leukocyte antigen.

The clinical grade of UMSC01 was certified and identified by surface markers, which showed uniformly high expression (≥95%) of CD73, CD90, and CD105 but low expression (≤2%) of CD11b, CD34, CD45, CD19, and HLA-DR (Figure 2A). The capacity of the potency assay for *in vitro* differentiation into mesodermal lineages of adipocytes, chondrocytes, and osteocytes was confirmed by Oil-Red-O, Alcian-Blue and Alizarin-Red staining, respectively (Figure 2B). The final drug substance and drug product of UMSC01 were released after passing all the criteria including cell viability, microbiological tests, endotoxin level, and Gram staining.

### Primary endpoints for safety

There were no significant changes in the blood pressures, heart rates, or oxygenation status before and after IC injection or IV infusion of UMSC01 (Supplementary Figure S1). The culprit arteries remained patent at the end of IC infusion in all subjects, and a steady decrease was seen in troponin-I (18.05 ± 27.46 vs. 1.66 ± 1.49 ng/L, *p* = 0.0313) and creatine kinase-MB (CK-MB; 30.7 ± 61.3 vs. 1.9 ± 1.0 ng/L, *p* = 0.0313) levels at 24 h compared to the baseline values (graphical abstract). The FEV1% was comparable (81.17 ± 5.74 vs. 84.83 ± 14.52%, *p* = 0.6875) at the end of IV infusion of UMSC01 (Graphical Abstract). There were significant differences in the levels of blood urea nitrogen (14 ± 3 vs. 19 ± 2 mg/dL, *p* = 0.0313), serum potassium (3.6 ± 0.2 vs. 4.2 ± 0.2 meq/L, *p* = 0.0313), white blood cell count (9.9 ± 2.9 vs. 6.5 ± 2.1 10^3^/μL, *p* = 0.0313), platelet count (204 ± 32 vs. 227 ± 38 10^3^/μL, *p* = 0.0313), and the plasma IgG (1050 ± 292 vs. 1225 ± 342 mg/dL, *p* = 0.0313) between baseline and 12-month follow-up, however these values were within normal ranges and were not associated with any clinically relevant events (Table 2). The carcinoembryonic antigen level (4.27 ± 4.19 vs. 2.71 ± 1.22 ng/mL, *p* = 0.2188) and immunology parameters including CD3 (55.3 ± 12.3 vs. 58.9 ± 6.1%, *p* = 0.4375), CD4/CD8 (2.8 ± 1.2 vs. 2.1 ± 0.8, *p* = 0.0938), anti-HLA antibodies (0.6 ± 0.4 vs. 0.6 ± 0.7%, *p* = 0.7500), and panel reactive antibody assay (0.4 ± 0.4 vs. 0.4 ± 0.5%, *p* = 0.6250) were not significantly different between baseline and at the 12-month follow-up. During the study period, none of the subjects experienced serious adverse events or major adverse cardiovascular events (Table 3). One patient had a unilateral inguinal hernia at 5-month of follow-up and was subsequently hospitalized for operation, which was adjudicated as a non-treatment related serious adverse event. There were three non-treatment related adverse events consisting of a non-ischemic chest pain, a mild superficial skin rash, and a localized eczema, respectively in three subjects.

**TABLE 2:** Serial laboratory tests during the study period.

	Case 1	Case 2	Case 3	Case 4	Case 5	Case 6	Mean ± SD	*P*-value^a^
**AST (IU/L)**								
Baseline	109	26	36	67	41	53	55 ± 30	–
After IC injection	58	16	20	18	24	20	26 ± 16	**0.0313**
After IV infusion	56	17	26	19	13	17	25 ± 16	**0.0313**
1-Month follow-up	52	19	23	18	19	21	25 ± 13	**0.0313**
3-Month follow-up	66	16	27	20	17	19	28 ± 19	**0.0313**
6-Month follow-up	30	23	21	28	15	29	24 ± 6	**0.0313**
12-Month follow-up	39	32	25	17	16	15	24 ± 10	**0.0625**
**ALT (IU/L)**								
Baseline	122	14	15	29	54	25	43 ± 41	–
After IC injection	91	20	17	25	48	24	38 ± 28	**0.4688**
After IV infusion	95	20	23	23	31	22	36 ± 29	**0.4688**
1-Month follow-up	82	14	25	16	41	27	34 ± 25	**0.2500**
3-Month follow-up	81	14	33	18	26	25	33 ± 25	**0.3750**
6-Month follow-up	40	33	19	35	19	36	30 ± 9	**1.0000**
12-Month follow-up	55	27	18	12	14	16	24 ± 16	**0.2188**
**BUN (mg/dL)**								
Baseline	14	10	12	13	19	14	14 ± 3	**−**
After IC injection	12	11	12	16	16	10	13 ± 3	**0.5000**
After IV infusion	–	11	13	17	11	10	12 ± 3	**0.9375**
1-Month follow-up	26	10	13	13	16	19	16 ± 6	**0.3750**
3-Month follow-up	16	15	14	19	22	16	17 ± 3	**0.0313**
6-Month follow-up	18	12	12	20	22	16	17 ± 4	**0.0625**
12-Month follow-up	20	19	16	21	20	18	19 ± 2	**0.0313**
**Creatinine (mg/dL)**								
Baseline	0.77	0.79	0.77	1.08	0.85	1.06	0.89 ± 0.15	**−**
After IC injection	0.72	0.93	0.82	0.98	0.83	1.09	0.90 ± 0.13	**0.9063**
After IV infusion	0.81	0.98	0.85	0.89	0.87	1.19	0.93 ± 0.14	**0.3438**
1-Month follow-up	1.01	1.09	1.04	1.25	0.95	1.25	1.10 ± 0.13	**0.0313**
3-Month follow-up	0.89	1.06	1.05	1.49	0.86	1.02	1.06 ± 0.23	**0.0938**
6-Month follow-up	0.89	0.93	0.95	1.31	0.84	0.97	0.98 ± 0.17	**0.1563**
12-Month follow-up	0.82	1.13	0.86	1.36	0.77	1.05	1.00 ± 0.23	**0.2188**
**eGFR (mL/min/1.73 m^2^)**								
Baseline	106	105	100	67	93	69	90 ± 18	**−**
After IC injection	114	87	93	75	96	67	89 ± 17	**0.9375**
After IV infusion	100	82	89	84	91	60	84 ± 13	**0.3125**
1-Month follow-up	77	72	71	57	82	57	69 ± 10	**0.0313**
3-Month follow-up	89	75	70	46	92	72	74 ± 16	**0.0938**
6-Month follow-up	89	87	78	54	95	77	80 ± 14	**0.1563**
12-Month follow-up	98	69	88	52	105	70	80 ± 20	**0.2500**
**Sodium (mmol/L)**								
Baseline	138	141	139	141	141	136	139 ± 2	**–**
After IC injection	137	139	139	141	139	139	139 ± 1	**0.8750**
After IV infusion	135	140	139	140	141	139	139 ± 2	**0.7500**
1-Month follow-up	139	143	140	142	142	139	141 ± 2	**0.0313**
3-Month follow-up	138	141	141	144	142	139	141 ± 2	**0.1250**
6-Month follow-up	139	141	142	141	142	140	141 ± 1	**0.1250**
12-Month follow-up	141	142	139	143	139	140	141 ± 2	**0.2500**
**Potassium (mmol/L)**								
Baseline	3.3	3.9	3.7	3.7	3.3	3.7	3.6 ± 0.2	**–**
After IC injection	3.5	3.8	3.8	3.4	3.5	4	3.7 ± 0.2	**0.5938**
After IV infusion	3.8	3.8	3.9	3.4	3.5	4.2	3.8 ± 0.3	**0.2813**
1-Month follow-up	3.6	4.4	3.5	3.7	3.8	3.9	3.8 ± 0.3	**0.1875**
3-Month follow-up	4.1	4.1	4.2	4	4.5	4.2	4.2 ± 0.2	**0.0313**
6-Month follow-up	3.6	4.1	3.9	3.8	3.9	4	3.9 ± 0.2	**0.0313**
12-Month follow-up	4.2	4.4	4.1	4.1	4.2	3.9	4.2 ± 0.2	**0.0313**
**RBC (10** ^6^ **/uL)**								
Baseline	5.14	4.67	6.87	4.79	4.89	4.57	5.16 ± 0.86	**–**
After IC injection	5.17	4.53	6.24	4.23	4.59	4.08	4.81 ± 0.80	**0.0625**
After IV infusion	5.48	4.57	6.10	4.37	4.47	4.14	4.86 ± 0.76	**0.0938**
1-Month follow-up	5.16	4.77	6.55	5.20	4.64	4.21	5.09 ± 0.80	**0.8438**
3-Month follow-up	5.04	4.57	6.44	4.88	4.87	3.84	4.94 ± 0.85	**0.0938**
6-Month follow-up	5.21	5.04	6.61	4.70	4.92	3.88	5.06 ± 0.89	**0.6875**
12-Month follow-up	5.07	4.79	6.58	4.40	4.79	4.07	4.95 ± 0.87	**0.1563**
**WBC (10** ^3^ **/μL)**								
Baseline	11.0	15.2	8.1	8.1	9.4	7.7	9.9 ± 2.9	**–**
After IC injection	7.2	11.1	6.4	6.8	9.9	6.8	8.0 ± 2.0	**0.0625**
After IV infusion	8.1	11.4	5.8	6.9	11.0	8.1	8.6 ± 2.2	**0.2188**
1-Month follow-up	7.4	7.2	5.1	5.9	7.8	5.1	6.4 ± 1.2	**0.0313**
3-Month follow-up	6.7	9.1	5.9	7.2	8.3	4.8	7.0 ± 1.6	**0.0313**
6-Month follow-up	6.7	8.0	5.5	5.8	6.7	6.9	6.6 ± 0.9	**0.0313**
12-Month follow-up	6.0	10.7	6.0	5.2	6.0	4.9	6.5 ± 2.1	**0.0313**
**Hemoglobin (g/dL)**								
Baseline	15.6	13.8	15.1	14.1	15.3	15.4	14.9 ± 0.7	**−**
After IC injection	15.9	13.7	14.2	12.9	14.3	13.5	14.1 ± 1.0	**0.0938**
After IV infusion	16.7	13.4	13.9	13.4	14.0	13.8	14.2 ± 1.3	**0.1563**
1-Month follow-up	15.7	14.9	15.2	15.2	14.3	13.8	14.9 ± 0.7	**0.8750**
3-Month follow-up	15.3	13.7	14.6	13.7	14.9	12.5	14.1 ± 1.0	**0.0313**
6-Month follow-up	16.0	14.6	15.2	13.7	14.7	12.7	14.5 ± 1.2	**0.7500**
12-Month follow-up	15.7	15.0	15.3	13.1	14.8	13.0	14.5 ± 1.2	**0.6875**
**Platelet count (10** ^3^ **/μL)**								
Baseline	173	262	192	180	209	205	204 ± 32	**–**
After IC injection	204	467	211	161	214	203	243 ± 111	**0.2500**
After IV infusion	232	517	231	174	233	229	269 ± 123	**0.0625**
1-Month follow-up	172	293	193	189	224	247	220 ± 45	**0.0938**
3-Month follow-up	175	313	204	324	251	253	253 ± 59	**0.0313**
6-Month follow-up	182	352	238	230	216	263	247 ± 58	**0.0313**
12-Month follow-up	195	301	224	202	212	227	227 ± 38	**0.0313**
**Carcinoembryonic antigen (ng/mL)**								
Baseline	3.16	3.69	1.84	3.37	1.00	12.57	4.27 ± 4.19	**–**
After IC injection	2.86	2.63	2.15	2.46	0.98	10.71	3.63 ± 3.53	**0.1563**
After IV infusion	3.67	2.64	1.84	3.28	0.99	9.84	3.71 ± 3.16	**0.3125**
1-Month follow-up	2.26	3.22	1.80	2.32	0.82	7.18	2.93 ± 2.22	**0.0313**
3-Month follow-up	2.44	3.18	1.85	4.02	1.34	5.44	3.05 ± 1.51	**0.5625**
6-Month follow-up	2.77	2.69	2.41	2.45	0.79	4.17	2.55 ± 1.08	**0.1563**
12-Month follow-up	3.20	3.31	2.10	2.23	0.95	4.48	2.71 ± 1.22	**0.2188**
**CK (IU/L)**								
Baseline	2163	289	331	610	186	335	652 ± 753	**−**
24 h after IC injection	231	47	80	129	123	118	121 ± 62	**0.0313**
24 h after IV infusion	128	42	94	70	83	96	86 ± 29	**0.0313**
Discharge	150	38	107	79	62	69	84 ± 39	**0.0313**
**CD3 (%)**								
Baseline	52.6	76.8	49.8	41.1	50.6	60.6	55.3 ± 12.3	**−**
After IC injection	60.5	59.7	−	42.3	−	43.7	51.6 ± 9.9	**0.6250**
After IV infusion	58.9	−	39.5	36.8	64.7	68.1	53.6 ± 14.5	**0.6250**
1-Month follow-up	58.6	55.7	60.7	41.0	60.3	74.1	58.4 ± 10.6	**0.5625**
3-Month follow-up	60.8	67.1	48.6	34.8	54.8	63.1	54.9 ± 11.8	**1.0000**
6-Month follow-up	69.3	63.7	54.4	52.1	54.6	64.0	59.7 ± 6.9	**0.3125**
12-Month follow-up	57.5	64.8	56.5	48.9	60.6	65.3	58.9 ± 6.1	**0.4375**
**CD4/CD8**								
Baseline	2.1	1.8	4.0	3.4	4.1	1.2	2.8 ± 1.2	**–**
After IC injection	1.8	2.0	−	3.4	−	1.9	2.3 ± 0.8	**0.6250**
After IV infusion	1.6	−	5.1	2.5	3.7	1.7	2.9 ± 1.5	**1.0000**
1-Month follow-up	1.6	1.3	4.4	2.3	4.4	1.1	2.5 ± 1.5	**0.3125**
3-Month follow-up	1.5	1.6	4.1	2.0	4.0	0.8	2.3 ± 1.4	**0.0625**
6-Month follow-up	1.7	1.5	3.0	2.6	2.6	0.6	2.0 ± 0.9	**0.0313**
12-Month follow-up	2.0	1.4	3.4	2.4	2.0	1.2	2.1 ± 0.8	**0.0938**
**IgG (mg/dL)**								
Baseline	759	1100	1430	1020	689	1300	1050 ± 292	**–**
After IC injection	931	1050	1490	806	677	922	979 ± 280	**0.5625**
After IV infusion	967	1210	1440	930	649	1380	1096 ± 302	**0.4375**
1-Month follow-up	970	1120	1530	1180	932	1760	1249 ± 329	**0.0313**
3-Month follow-up	892	1250	1650	1270	943	1570	1263 ± 311	**0.0313**
6-Month follow-up	917	1210	1790	1060	914	1510	1234 ± 352	**0.0313**
12-Month follow-up	823	1180	1740	1160	947	1500	1225 ± 342	**0.0313**
**IgM (mg/dL)**								
Baseline	208.0	140.0	72.5	97.4	30.1	44.9	98.8 ± 66.2	**–**
After IC injection	226.0	169.0	71.0	94.1	19.1	25.2	100.7 ± 82.0	**1.0000**
After IV infusion	272.0	174.0	70.3	105.0	20.6	36.2	113.0 ± 95.2	**0.6875**
1-Month follow-up	235.0	160.0	74.2	129.0	24.6	47.0	111.6 ± 78.8	**0.1563**
3-Month follow-up	220.0	146.0	75.4	161.0	28.9	41.8	112.2 ± 75.4	**0.2188**
6-Month follow-up	208.0	133.0	82.2	110.0	18.9	37.8	98.3 ± 68.7	**1.0000**
12-Month follow-up	181.0	116.0	80.5	119.0	21.7	37.9	92.7 ± 58.7	**0.4375**
**Anti-HLA antibodies (%)**								
Baseline	0.7	0.2	1.0	0.4	0.4	1.1	0.6 ± 0.4	**–**
After IC injection	1.9	0.7	0.5	0.1	0.3	0.5	0.7 ± 0.6	**0.9063**
After IV infusion	1.7	0.4	0.7	0.3	3.9	0.4	1.2 ± 1.4	**0.6875**
1-Month follow-up	2.4	0.9	2.4	1.7	1.6	0.1	1.5 ± 0.9	**0.0938**
3-Month follow-up	0.7	0.1	0.8	0.7	0.1	0.6	0.5 ± 0.3	**0.3750**
6-Month follow-up	0.8	1.9	0.7	1.0	0.5	0.9	1.0 ± 0.5	**0.5625**
12-Month follow-up	0.3	0.2	1.9	0.5	0.1	0.8	0.6 ± 0.7	**0.7500**
**Panel reactive antibody assay (%)**								
Baseline	0.40	0.20	0.30	0.20	0.30	1.20	0.4 ± 0.4	**–**
After IC injection	1.20	0.10	0.40	0.20	0.40	0.70	0.5 ± 0.4	**0.8125**
After IV infusion	1.00	0.10	0.40	0.10	0.50	0.10	0.4 ± 0.4	**1.0000**
1-Month follow-up	0.60	0.30	1.50	0.90	0.50	0.10	0.7 ± 0.5	**0.3125**
3-Month follow-up	0.20	0.10	0.20	0.20	0.10	0.50	0.2 ± 0.1	**0.0625**
6-Month follow-up	0.30	0.70	0.50	1.20	0.40	0.80	0.7 ± 0.3	**0.3438**
12-Month follow-up	0.10	0.10	1.30	0.20	0.10	0.30	0.4 ± 0.5	**0.6250**

^a^Wilcoxon signed-rank test.

ALT, alanine aminotransferase; AST, aspartate aminotransferase; BUN, blood urea nitrogen; CD, cluster of differentiation; CK, creatine kinase; eGFR, estimated glomerular filtration rate; HLA, human leukocyte antigen; Ig, immunoglobulin; IC, intracoronary; IV, intravenous; RBC, red blood cell; WBC, white blood cell; SD, standard deviation.

**TABLE 3:** Adjudication of treatment related adverse events among study subjects.

	Case 01	Case 02	Case 03	Case 04	Case 05	Case 06	*n* (%)
**SUSAR** ^a^	N	N	N	N	N	N	0 (0.00)
**SAE**							
Death	N	N	N	N	N	N	0 (0.00)
Life-threatening^b^	N	N	N	N	N	N	0 (0.00)
Hospitalization^c^	N	N	N	N	N	**Y**	1 (16.67)
Disability/Incapacity	N	N	N	N	N	N	0 (0.00)
Congenital anomaly/Birth defect	N	N	N	N	N	N	0 (0.00)
**AE** ^d^							
Chest pain	N	N	N	N	N	**Y**	1 (16.67)
*Severity*	–	–	–	–	–	*Mild*	
*Relationship with study IP*	–	–	–	–	–	*Unrelated*	
Eczema	N	**Y**	N	N	N	N	1 (16.67)
*Severity*	–	*Mild*	–	–	–	–	
*Relationship with study IP*	–	*Unrelated*	–	–	–	–	
Inguinal hernia	N	N	N	N	N	**Y**	1 (16.67)
*Severity*	–		–	–	–	*Moderate*	
*Relationship with study IP*	–		–	–	–	*Unrelated*	
Rash	N	N	N	N	**Y**	N	1 (16.67)
*Severity*	–	–	–	–	*Mild*	–	
*Relationship with study IP*	–	–	–	–	*Unrelated*	–	
**MACE**	N	N	N	N	N	N	0 (0.00)

^a^The nature and severity of which is not consistent with the applicable drug information.

^b^The subject was at risk of death at the time of event.

^c^The subject required hospitalization or prolonged existing hospitalization.

^d^Any untoward medical occurrence in a patient or clinical investigation subject administered a harmaceutical drug and which did not necessarily have to have a causal relationship with this treatment.

AE, adverse event; MACE, major adverse cardiovascular events; IP, investigational product; SAE, serious adverse event; SUSAR, suspected and unexpected serious adverse reaction; TEAE, treatment emergent adverse event.

### Secondary endpoints for efficacy

**FIGURE 3:**
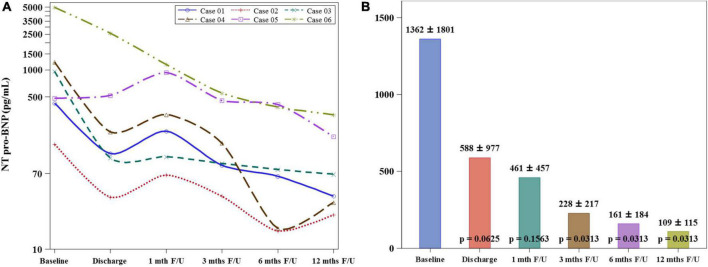
Comparison of NT pro-BNP levels between baseline and 12-month follow-up. **(A)** The serum level of NT pro-BNP of individual study patients shows a consistent declination pattern from baseline to 12-month follow-up. **(B)** The mean serum level of NT-proBNP decreased significantly at 12-month follow-up when compared to the value at baseline.

**FIGURE 4:**
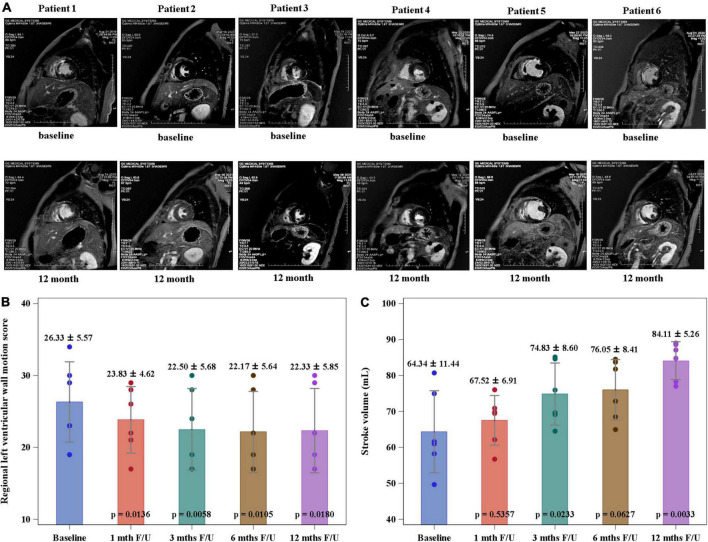
Regional left ventricular wall motion score (RLVWMS) and stroke volume evaluated by cardiac magnetic resonance imaging (CMRI). **(A)** The representative CMRIs of individual study patient at baseline and 12-month follow-up. The RLVWMS decreased **(B)** and the stroke volume increased **(C)** at 12-month follow-up compared to the baseline levels.

The mean serum level of NT-proBNP decreased significantly at 12-month follow-up when compared to the value at baseline (1362 ± 1801 vs. 109 ± 115 pg/mL, *p* = 0.0313) (Figure 3). The stroke volume increased from 64.34 ± 11.44 ml/per beat to 84.11 ± 5.26 ml/per beat (*p* = 0.0033) and LVEF from 52.67 ± 12.75% to 62.47 ± 17.35% (*p* = 0.0246), while the wall motion score decreased from 26.33 ± 5.57 to 22.33 ± 5.85 (*p* = 0.0180) at the 12-month follow-up compared to the baseline levels by CMRI (Graphical Abstract and Figure 4). The New York Heart Association functional classification improved from class II at baseline to class I at 12-month follow-up in all study subjects.

## Discussion

We report a phase I clinical trial of combined IC and IV delivery of UMSC01 that reached the specified endpoints: STEMI patients safely received cell transplantation in this dual-route administration until the 12-month follow-up. To the best of our knowledge, this is the first-in-human study to demonstrate the safety and feasibility of a combined delivery method in AMI patients with STEMI and heart failure. Under the current study design, we observed an improvement in LVEF and functional status, which encourages a randomized double-blind phase II study.

### Strengths of UMSC01

To explore the appropriate human MSCs for clinical applications, pluripotent-like markers for culturing MSCs that retain potent survival and self-renewal abilities should be thoroughly investigated. In our previous preclinical report (23), an insulin-like growth factor 1 receptor (IGF1R) expressing sub-population in human MSCs, including umbilical cord-MSCs, were cultured in platelet-derived growth factor-BB (PDGF-BB)-containing human umbilical cord serum (hUCS) and displayed longer survival and stronger proliferation potential. Through intercellular receptor transactivation between CXCR4 and IGF1R signaling pathways, implantation of IGF1R^+^ MSCs showed significant improvement in neurological function in a stroke model. In this clinical study, we translated the experimental setting by replacing the hUCS with commercially available platelet rich plasma containing high levels of PDGF-BB for culturing UMSC01 to treat the STEMI patients. The preliminary result found that UMSC01 administration might provide not only a safe but also a functional improvement strategy for STEMI patients in this pilot study. A larger-scale placebo-controlled trial is mandatory for demonstrating the definite clinical efficacy in the future.

In patients with AMI, a recent meta-analysis study demonstrated that transplantation of MSCs significantly improves left ventricular ejection fraction (LVEF) (24). Although Both BMNCs and MSCs contribute to an improvement of cardiac function in the clinical setting of AMI, it is important to know which type of stem cell could outperform the other (25). So far, there is no clinical trial with head-to-head comparison in evaluating the clinical efficacy between BMNCs and MSCs in patients with AMI. Interestingly, Hosseinpour et al. 25 reported that transplantation of MSCs might result in better LVEF improvement than BMNCs in a meta-analysis study. Among the different types of MSCs, umbilical cord-derived MSCs can be easily obtained and cultured (26, 27). Umbilical cord-derived MSCs have shown immunomodulatory and tissue-repair effects with low immunogenicity, which makes them ideal candidates for allogeneic adoptive transfer therapy (28, 29).

### Mode of delivery

Stem cell-based cardiac therapy involves the transplantation of cells via various delivery methods. Currently, there are three main routes of cell implantation—intramyocardial (transepicardial or transendocardial), IC, and IV (7). Each delivery technique aims to transfer an adequate number of cells to the infarct site of the heart and to maximize the retention rate of cells, thus improving engraftment and facilitating robust therapeutic outcomes (30). Nevertheless, irrespective of the route of cell delivery, low retention remains a major hurdle limiting the beneficial effects of cell transplantation (31). To date, the vast majority of animal and clinical studies on stem cell therapy in cardiovascular disease have chosen a single delivery method. It is possible that combined IC and IV stem cell transplantation provides more benefits than IC or IV delivery alone (21). The combination of IC and IV for cell delivery, like IC alone, increases the number of cells homing to the injured area of the myocardium, while subsequent IV stem cell treatment allows for a higher cell dose and also exerts systemic anti-inflammatory effects to achieve better therapeutic outcome. Importantly, a study conducted by Liu et al. used a combined approach of cell delivery (IC injection with IV infusion) in a porcine model of chronic myocardial ischemia (32). Their results showed that umbilical cord-MSCs improved the left ventricular function, perfusion, and remodeling. In addition, there was a significant reduction in fibrosis and apoptosis (32). In our study design, we were able to deliver a total of 10^8^ UMSC01 uneventfully. There was no any treatment-related adverse events, including coronary occlusion, immune reaction, or tumor formation. As a pilot phase one study, it is not possible to draw a solid conclusion regarding the efficacy of UMSC01 transplantation. Nevertheless, another randomized clinical trial is ongoing, which compares single with double IC infusion of umbilical cord-derived Wharton’s jelly MSCs in patients with AMI (33). The result may provide better understanding of the effect of repeated transplantation of this cell type on myocardial function (LVEF).

### Timing of delivery

Following STEMI, the key events include an acute inflammatory phase in the first 4 days, resolution and repair phase within 2 weeks, and a remodeling phase after 2 weeks (34, 35). Blunting the infarct-triggered inflammation and promotion of late healing are important for the reduction of abnormal cardiac remodeling (36–38). It has been extensively studied in previous studies regarding time of delivery in stem cell therapy after AMI (39–43). For BMNCs, it was suggested to deliver stem cells 3–7 days after AMI for improvement of myocardial function (24). Consistently, a meta-analysis also shows a higher efficacy if MSCs transplantation was performed within the first week following AMI (24). In our study, IC treatment on the 4–5th day post-MI ensured a stable post-AMI condition and was able to modulate the acute immune response and shape acute inflammation. The IV cell administration on the 6–7th day post-MI improved the myocardial repair process and modified scar replacement. A longer stem cell treatment period that covered the golden first 2 weeks after AMI potentially augmented the regeneration effect.

### Cell therapy for acute myocardial infarction in post-BAMI era

In most stem cell-treated AMI trials, transplanted cells were BMNCs and the benefits in cardiac regeneration were mixed and uncertain (10). To date, the autologous bone marrow cell therapy in AMI trial (BAMI) is the largest study of stem-based cell therapy for AMI in the world. Initially, BAMI was designed to establish whether BMNCs reduced mortality in patients with STEMI by IC infusion. Unfortunately, the low number of cases was unable to show a significant difference in improving survival between patients and the case–control group. In contrast to BMNCs, MSCs show consistent findings of improvement in cardiac function. MSCs, especially UMSC01, have multiple differentiation capabilities, immune exemption, easy access, large-scale expansion, and ethical advantages. The work by Gao et al. (29) demonstrated that IC delivery of UMSC01 could significantly improve myocardial viability and cardiac function in patients with STEMI. Furthermore, in our study, we showed that UMSC01 delivery using this novel administration method is safe and feasible in human beings. Although we did not include a control group in this trial, the enrolled patients showed an improvement in cardiac function, regional wall motion, heart failure symptoms, and biomarkers, indicating that UMSC01 transplantation might be beneficial in post-AMI cardiac repair.

Over the last two decades, almost 100 blinded and unblinded AMI clinical trials worldwide have shown an excellent safety profile of cell-based therapy but have reported mixed and uncertain results on its potential benefits (9, 10, 21, 44). Of note, the number of enrolled patients in many of the clinical trials were not sufficient to test the benefits of stem cell transplantation when combined with the current guideline-directed AMI therapy (44, 45). The BAMI trial failed to demonstrate that BMNC therapy improves survival in patients with AMI due to low enrollment and low mortality rates. However, the BAMI researchers observed the clinical benefit of reduced heart failure associated hospitalization in patients receiving BMNC therapy (45). In a recent meta-analysis study, Attar et al. further confirmed that BMNC therapy improved clinical outcomes in terms of reinfarction and hospitalization for heart failure (46). Thus, it is crucial to identify a better cell type, optimal dose, and route of transplantation to strengthen the therapeutic effect and achieve a statistical significance in the post-BAMI era (10).

### Study limitations

This study had some limitations. First, although this pilot trial was designed to determine whether the novel approach of combined IC and IV delivery of UMSC01 in STEMI patients is safe, the sample size of six analyzable subjects was relatively small. Second, placebo-treated patients were not included in this phase I study. The efficacy of UMSC01 transplantation remains unclear. However, stem cell-treated patients exhibited improvement in heart function after a 12-month follow-up period. Third, this study focused mainly on the safety and feasibility of the combined IC and IV methods and thus did not provide comprehensive multimodality imaging in the assessment of efficacy. Lastly, in this pilot study, although we observed no significant changes of several immunology parameters including CD3, CD4/CD8, anti-HLA antibodies, and panel reactive antibody assay between baseline and at the 12-month follow-up, it is indeed necessary to measure more immunology/inflammatory markers such as IL-6 and IL-10 in the future phase II study.

## Conclusion

In this pilot study, our approach of IC injection combined with IV infusion of UMSC01 in STEMI patients with impaired LVEF appears to be safe, feasible, and potentially beneficial in improving cardiac systolic function and heart failure symptoms up to 12 months after treatment. As this is the first trial of dual-route transplantation of stem cells in humans, larger randomized and placebo-controlled phase II studies are required to demonstrate the efficacy of this novel approach.

## Data availability statement

The original contributions presented in this study are included in the article/Supplementary material, further inquiries can be directed to the corresponding authors.

## Ethics statement

The studies involving human participants were reviewed and approved by the Research Ethics Committee of China Medical University Hospital (CMUH107-REC1-088). The trial was monitored by an independent data and safety monitoring board who met as planned to assess adverse events. The patients/participants provided their written informed consent to participate in this study.

## Author contributions

L-CH, Y-NL, and W-CS: conception and design, provision of study material or patients, data analysis and interpretation, manuscript writing, and final approval of manuscript. MH, C-RL, S-SC, Y-CW, J-YC, S-YL, K-YL, and Y-KL: provision of study material or patients, collection of data, data analysis and interpretation, and final approval of manuscript. M-YW, W-YT, M-YS, C-TH, C-KT, L-TC, C-LC, C-LL, and K-CH: data analysis and interpretation, final approval of manuscript. D-YC and C-HT: financial support and administrative support. K-CC and L-BJ: financial support, conception and design, provision of study material or patients, data analysis and interpretation, manuscript writing, and final approval of manuscript. All authors contributed to the article and approved the submitted version.

## Abbreviations

ACC: American College of Cardiology
AE: adverse event
AHA: American Heart Association
AMI: acute myocardial infarction
BMNC: bone marrow mononuclear cell
CD: cluster of differentiation
CEA: carcinoembryonic antigen
CK-MB: creatine kinase-myocardial band
CMRI: cardiac magnetic resonance imaging
CXCR4: C-X -C Motif Chemokine Receptor 4
ECG: electrocardiography
FEV1: forced expiratory volume in 1 s
HLA: human leukocyte antigen
hUCS: human umbilical cord serum
IABP: intra-aortic balloon pump
IC: intracoronary
ICU: intensive care unit
Ig: immunoglobulin
IGF1R: insulin-like growth factor 1 receptor
IV: intravenous
LVEF: left ventricular ejection fraction
MACE: major adverse cardiovascular events
MOLLI: modified look-locker inversion recovery
MSC: mesenchymal stem cell
NT pro-BNP: amino-terminal pro-brain natriuretic peptide
PCI: percutaneous coronary intervention
PDGF-BB: platelet-derived growth factor-BB
RLVWMS: regional left ventricular wall motion score
SAE: serious adverse event
STEMI: ST-elevation myocardial infarction
SUSAR: suspected and unexpected serious adverse reaction
TIMI: thrombolysis in myocardial infarction
UC-MSC: umbilical cord-derived mesenchymal stem cell.
