# Supplementary material for: First-in-human pilot trial of combined intracoronary and intravenous mesenchymal stem cell therapy in acute myocardial infarction
Source: Front Cardiovasc Med. 2022 Aug 9;9:961920. doi: 10.3389/fcvm.2022.961920 (PMC9395611; doi:10.3389/fcvm.2022.961920)
Supplement: Supplementary file 1 [file Data_Sheet_1.docx]

**First-in-Human Pilot Trial of Combined Intracoronary and Intravenous Mesenchymal Stem Cell Therapy in Acute Myocardial Infarction**

Lien-Cheng Hsiao, MD, PhD, **^a, b*^**, Yen-Nien Lin, MD, PhD **^a, b*^**, Woei-Cherng Shyu, MD, PhD **^c, d*^**, Ming Ho, MD**^e^**, Chiung-Ray Lu, MD**^a^**, Shih-Sheng Chang, MD, PhD**^a, b^**, Yu-Chen Wang, MD, PhD**^a, b^**, Jan-Yow Chen, MD, PhD**^a, b^**, Shang-Yeh Lu, MD**^a^**, Mei-Yao Wu MD, PhD**^f, g^**, Keng-Yuan Li**^a^**, Yu-Kai Lin, MD**^a^**, Wen-Yih I. Tseng, MD, PhD**^h^**, Mao-Yuan Su, PhD**^h^**, Chin-Ting Hsu, MS**^d^**, Cheng-Kang Tsai, MS**^d^**, Lu-Ting Chiu, MS**^d^**, Chien-Lin Chen, PhD**^d^**, Cheng-Li Lin, MS**^i, j^**, Kai-Chieh Hu, MS**^i, j^**, Der-Yang Cho, MD**^b, k^**, Chang-Hai Tsai, MD, PhD**^l^**, Kuan-Cheng Chang, MD, PhD**^a, b^**, and Long-Bin Jeng, MD**^m^**

*Drs Hsiao, Lin, and Shyu contributed equally to this work.

**^a^**Division of Cardiovascular Medicine, Department of Medicine, China Medical University Hospital, Taichung 404332, TAIWAN

**^b^**Graduate Institute of Biomedical Sciences, China Medical University, Taichung 404333, TAIWAN

**^c^**Department of Neurology and Translational Medicine Research Center, China Medical University Hospital, Taichung, 404332, TAIWAN

**^d^**Ever Supreme Bio Technology Co., LTD, Taichung, 403409, TAIWAN

**^e^**Department of Gynecology and Obstetrics, China Medical University, Taichung 404332, TAIWAN

**^f^**School of Post-Baccalaureate Chinese Medicine, China Medical University, Taichung 404333, TAIWAN

**^g^**Department of Chinese Medicine, China Medical University Hospital, Taichung 404332, TAIWAN

**^h^**Molecular Imaging Center, National Taiwan University, Taipei 10672, TAIWAN

**^i^**Management office for Health Data, China Medical University Hospital, Taichung 40447, TAIWAN

**^j^**College of Medicine, China Medical University, Taichung 404333, TAIWAN

**^k^**Department of Neurosurgery, China Medical University, Taichung 404333, TAIWAN

**^l^**Department of Pediatrics, China Medical University, Taichung 404333, TAIWAN

**^m^**Organ Transplantation Center, China Medical University Hospital, Taichung 40447, TAIWAN

**Supplementary methods**

**Study design and patients-inclusion and exclusion criteria**

Patients had to meet the inclusion criteria as follows: (1) Male or female, aged between 20 and 76 years on the date of consent; (2) Patients who presented with typical ischemic chest pain within 12 hours of symptom onset and were diagnosed with acute STEMI according to the 2013 American College of Cardiology (ACC) Foundation/American Heart Association (AHA) guidelines for the Management of STEMI; (3) Patients who underwent standard-of-care treatment for STEMI, where the immediate reperfusion management should include primary PCI and adjunctive antithrombotic therapy within 12 hours after symptom onset; (4) Patients who underwent successful acute reperfusion therapy (residual stenosis visually < 50%) and thrombolysis in myocardial infarction (TIMI) flow ≥ 2 with placement of an IC stent and have a patent infarct-related artery that is suitable for cell infusion to the target area of abnormal wall motion following myocardial infarction; (5) Patients with LVEF ≥ 30% and < 50% determined by an echocardiogram according to the standard procedure as previously reported;^1^ (6) Patients who were willing to sign the informed consent or assent by the next of kin; (7) Patients with stable vital signs for at least 48 hours, defined as normal respiration, afebrile, systolic pressure ≥ 90 mmHg and < 180 mmHg, heart rate > 50/min and < 110/min; (8) Adequate pulmonary function test defined as a forced expiratory volume in 1 second (FEV1) > 50% predicted and peripheral artery oxygen saturation ≥ 95% at room air; and (9) All male patients and female patients with child-bearing potential (between puberty and 2 years after menopause) should use appropriate contraception method(s). The exclusion criteria were the following: (1) Patients with cardiogenic shock (defined as systolic blood pressure < 80 mmHg requiring vasopressors, intra-aortic balloon pump [IABP] or extracorporeal membrane oxygenation); (2) Patients with severe aortic stenosis or regurgitation according to the recommendation of the 2014 AHA/ACC guideline for the Management of Patients with Valvular Heart Disease; (3) Patients with severe mitral stenosis or regurgitation according to the recommendation of the 2014 AHA/ACC guideline for the Management of Patients with Valvular Heart Disease; (4) Patients who require staged PCI or coronary artery bypass graft surgery; (5) Patients with immuno-compromised conditions, known autoimmune conditions, or receiving immunosuppressive treatments; (6) Patients who are unable to undergo CMRI scan for any reason; (7) Patients with inadequate hepatic and renal function after the onset of AMI, defined as aspartate aminotransferase and alanine aminotransferase ≥ 4 × upper limit of normal and estimated glomerular filtration rate < 30 mL/min, respectively; (8) Patients with a history of malignant tumor or other clinically significant cardiovascular diseases that will confound the results of this study; (9) Patients who participated in other clinical trials in the last 3 months; and (10) Patients not suitable to participate in this trial as judged by the investigator(s).

**Preparation of UMSC01**

The umbilical cord from an eligible donor who consented to its use by UMSC01 production was cut with a sterile scalpel to 1 cm in length after removing the blood vessels. The umbilical cord tissue was washed with Hank’s Balanced Salt Solution (Biological Industries), chopped into small pieces (~1 mm^3^), and digested using collagenase type I (2 mL/cm of cord length; Thermo Fisher) in an incubator at 37°C for 2 h. Dulbecco's phosphate-buffered saline was mixed with the umbilical cord explant and filtered using 100 μm cell strainers and centrifuged; the final cell pellet was resuspended in a culture medium (Biological Industries) supplemented with platelet rich plasma (UltraGRO^TM^-Advanced, AventaCell) and cultured on a CellBind-coated 25T Flask (Corning) in a 37°C, 5% CO_2_ incubator. Metabolic waste was removed and replenished every 3-4 d by replacing the growth medium. When the cells reached 95% confluence, they were detached using TrypLE (Thermo Fisher Scientific Inc.) and seeded in a hyperflask at a density of 2000-4000 cells/cm^2^ in a 5% CO_2_ incubator at 37°C for 2-4 passages (approximately 6-8 weeks) for the final harvest. Cells were washed and detached with TrypLE, and the cell pellet was resuspended at a cell density of ~1.0 × 10^7^ cells/mL in a 1:1 ratio of CS10 (Biolife Solution Inc., WA, USA) and albumin (CSL Behring). Some of the cells (drug substance) were subjected to microbiological tests (sterility test and mycoplasma examination). The final cell suspension was then cryopreserved in a liquid nitrogen cell tank for further clinical use.

**Laboratory tests and cardiac magnetic resonance imaging (Supplementary Methods)**

An immunoassay was conducted to monitor for immunogenicity of the product after transplantation, by testing for the following: CD3, CD4, CD8, IgM, IgG, anti-HLA antibodies, and panel reactive antibody assay. CEA tests were conducted to evaluate for tumorigenicity at baseline and each visit. Serum levels of NT-pro-BNP were tested prior to UMSC01 administration as baseline evaluation, prior to discharge from the hospital and at 1-, 3-, 6-, and 12-month follow-ups for efficacy evaluation. Pulmonary function tests were performed to monitor for potential adverse changes associated with treatment in the subjects.

Cardiac magnetic resonance imaging (CMRI) was performed using the 1.5-T Magnetom Aera (Siemens Healthcare, Erlangen, Germany) with a 30-channel cardiac coil array according to a standard operation procedure reported previously.^2^ Briefly, myocardial T1 mapping was performed using an ECG-triggered modified Look-Locker inversion recovery (MOLLI) pulse sequence before and 10 min after 0.15 mmol/kg IV administration of the gadolinium-based contrast agent (Dotarem, Guerbet, France). The MOLLI protocol used a 5(3)3 sampling scheme for native T1 mapping and a 4(1)3(1)2 sampling scheme for post-contrast T1 mapping. After post-contrast T1 acquisition, LGE images were obtained using an ECG-triggered phase-sensitive inversion recovery prepared segmented fast gradient-echo pulse sequence to identify focal fibrosis or scarring.

**Reference**

1. Cerqueira MD, Weissman NJ, Dilsizian V, et al. Standardized myocardial segmentation and nomenclature for tomographic imaging of the heart. A statement for healthcare professionals from the Cardiac Imaging Committee of the Council on Clinical Cardiology of the American Heart Association. Circulation 2002; 105(4): 539-42.

2. Beijnink CWH, van der Hoeven NW, Konijnenberg LSF, et al. Cardiac MRI to Visualize Myocardial Damage after ST-Segment Elevation Myocardial Infarction: A Review of Its Histologic Validation. Radiology 2021; 301(1): 4-18.

**Supplementary Figure S1.**

**
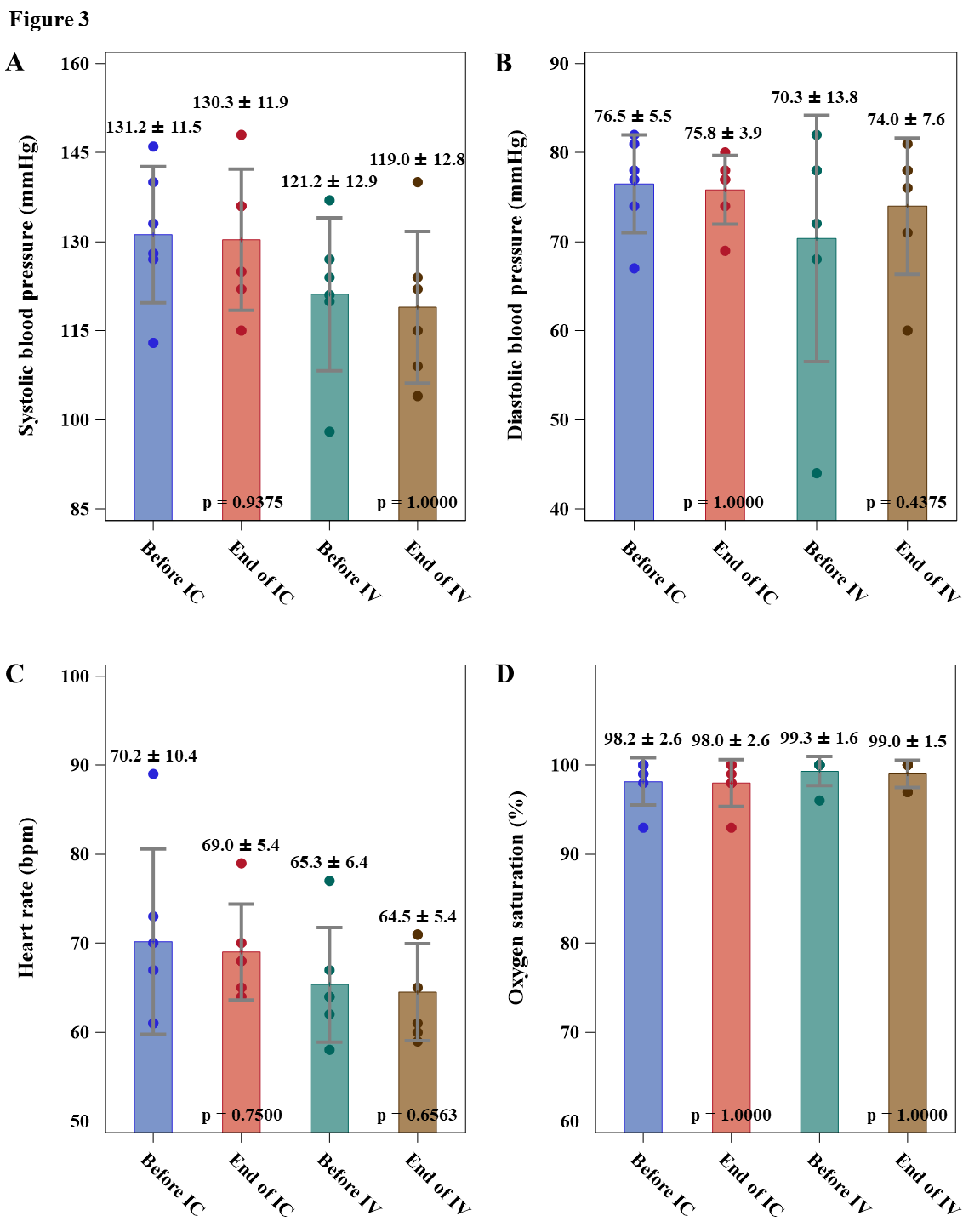
**

**Monitoring of Blood Pressure, Heart Rate, and Oxygen Saturation during Intracoronary (IC) or Intravenous (IV) Infusion of UMSC01.** There were no significant changes in systolic blood pressure **(A)**, diastolic blood pressure **(B)**, heart rate**(C)**, and oxygen saturation **(D)**, before and after IC or IV infusion of UMSC01. All comparisons were performed by Wilcoxon signed-rank tests.
